# Influence of *TP*53 Codon 72 Polymorphism Alone or in Combination with *HDM*2 SNP309 on Human Infertility and IVF Outcome

**DOI:** 10.1371/journal.pone.0167147

**Published:** 2016-11-29

**Authors:** Ying Chan, Baosheng Zhu, Hongguo Jiang, Jinman Zhang, Ying Luo, Wenru Tang

**Affiliations:** 1 Genetic Diagnosis Center, Yunnan Provincial Key Laboratory For Birth Defects and Genetic Diseases, The First People’s Hospital of Yunnan Province, Kunming, Yunnan, China; 2 Lab of Molecular Genetics of Aging & Tumor, Faculty of Medicine, Kunming University of Science and Technology, Kunming, Yunnan, China; Universitat des Saarlandes, GERMANY

## Abstract

To evaluate the association of the *TP*53 codon 72 (rs 1042522) alone or in combination with *HDM*2 SNP309 (rs 2279744) polymorphisms with human infertility and IVF outcome, we collected 1450 infertility women undergoing their first controlled ovarian stimulation for IVF treatment and 250 fertile controls in the case-control study. Frequencies, distribution, interaction of genes, and correlation with infertility and IVF outcome of clinical pregnancy were analyzed. We found a statistically significant association between *TP*53 codon 72 polymorphism and IVF outcome (52.10% vs. 47.40%, OR = 0.83, 95%CI:0.71–0.96, p = 0.01). No significant difference was shown between *TP*53 codon 72, *HDM*2 SNP309 polymorphisms, human infertility, and between **the combination of two genes polymorphisms** and the clinical pregnancy outcome of IVF. The data support C allele as a protective factor for IVF pregnancy outcome. Further researches should be focused on the mechanism of these associations.

## Introduction

Infertility is a global health problem with an estimated incidence of 10–15% of general reproductive age couples. The increasing couples of infertility are seeking for IVF-ET (in vitro fertilization-embryo transfer) treatment to be pregnant, but the main factors that determine the final outcome of treatment are still mostly undefined[1]. The most direct parameters associated with successful pregnancy contain maternal age, embryo quality and the endometrial receptivity. In addition, other causes leading to negative IVF outcome remain unknown. The trophoblastic invasion of blastocyst and establishment of angiogenesis involve in the procedure of embryo implantation, and these processes depend on the balance of growth and apoptosis [2]. Tumor suppressor gene P53 (*TP*53) was identified to be a potential inducer of apoptosis and angiogenesis [3, 4]. Recently, with the accumulation of human genomic knowledge and the development of DNA technology efficiency, genetic factors related to implantation failure can be explored generally, especially some single nucleotide polymorphisms (SNPs) identified in human[5].

*TP*53 is the most common mutated gene in human cancer researches and more than 50% of tumors exists mutations in this gene [6]. Tumor suppressor protein p53 is a transcription factor that can stop cell progression or promote apoptosis when cell suffers from stress signals[7]. The well-known functions of *TP*53 include tumor suppression, DNA damage repair, metabolic pathways, regulation of oxidative stress, invasion and motility, cellular senescence, angiogenesis, differentiation, bone remodeling and reproduction regulation. Recent studies suggested that *TP*53 participates in human reproduction mainly through regulating leukemia inhibitory factor (LIF) levels[8]. Otherwise, apoptosis and angiogenesis have played a crucial role during the normal pregnancy development [9]. *TP*53 codon 72 polymorphism (rs 1042522) is a vital functional polymorphic form which consists of either a wild C allele or a derived G allele. The C allele and G allele lead to a proline and an arginine in codon 72 location respectively. G allele shows a higher efficiency than C allele in view of inducing apoptosis, expression of leukemia inhibitory factor (LIF) and suppression of cell transformation [10]. Some studies revealed that *TP*53 codon 72 polymorphism is associated with human fertility, the higher frequency of C/C genotype distribution was found in recurrent pregnancy loss (RPL) than the other groups [11, 12], but it lacks a significant effect on implantation rate [13]. Kang et al. found *TP*53 codon 72 C allele is enriched in IVF patients with unexplained infertility [14], but the enrichment was not found by Patounakis et al.[15].

Human double minute 2 (*HDM*2; human ortholog of murine double minute 2) is a main negative regulator of p53[16]. TP53 and HDM2 form an autoregulatory negative feedback loop within which HDM2 negatively regulates TP53 levels and activity and TP53 positively regulates HDM2 levels[17]. An important polymorphism in the *HDM*2 gene at the 309th nucleotide was identified and results from a T to G change. The *HDM*2 SNP309 G/G (rs 2279744) polymorphism with raised promoter recognition by Sp1 transcription factor elevates *HDM*2 expression and attenuation of apoptosis mediated by *TP*53 [18]. It was reported that *HDM*2 SNP 309 polymorphism alone or in combination with *TP*53 codon 72 polymorphism may be related to missed abortion [19, 20].

Determining the roles of *TP*53 codon 72 and *HDM*2 SNP309 polymorphisms in human infertility and IVF pregnancy outcome is very important for the previous contradictory reports. The aim of the presented study was to define the possible impact of *TP*53 codon 72 polymorphism and/or *HDM*2 SNP 309 on human fertility and the first IVF pregnancy outcomes.

## Materials and Methods

### Subjects

All participants were recruited from The First People’s Hospital of Yunnan Province. Blood samples were collected from July 2010 to December 2012. Informed consents were signed by all participants before the procedure and all of them agreed to provide their samples for molecular study. The study was approved by The Ethics Review Board of The First People’s Hospital of Yunnan Province. The study was carried out in accordance with the Declaration of Helsinki and Good Clinical Practice guidelines. The sample size was evaluated for the genotyped SNPs with the use of a relevant genotype frequency of 45% in Asians, aiming at reaching 80% power to test an OR value of 1.5 for the case group. The research was ceased once it reached the statistical significance. The identifying information of patient was not included in the data analysis to protect patient privacy and anonymity.

Totally, 1450 IVF patients and 250 normal fertile control women were included in the study. Among the 1450 women undergoing the first IVF treatment, one case was excluded from analysis of polymorphisms and IVF outcome for the loss of follow-up. All subjects (age<40 years) enrolled in the study were normal according to their physical examination results and ruled out abnormal chromosome karyotype. The control group consists of 250 women without infertility history, who had delivered at least one child without any difficulties. The infertility group consists of 1450 patients undergoing their first IVF cycle, the exclusion criteria are as follows: 1) non-male factor infertility, 2) without normal pregnancy history, 3) cycles with Controlled Ovary Hyperstimulation (COH) and qualified embryo transfer. Tubal factor, mild endometriosis, ovulation failure, cervical factor, polycystic ovary syndrome and unexplained infertility constitute the pathogenesis of IVF patients in the study.

All patients undergoing IVF treatment were submitted to conventional COH using recombinant human FSH and pituitary suppression with GnRh antagonist. Ovulation was injected by 10000 recombinant hCG when at least three leading follicles had reached more than 17 mm, 36 hours later; follicle aspiration was performed under transvaginal ultrasound guidance. According to the standard IVF procedure, treatment of gametes, fertilization, embryo culture, embryo freezing and embryo transfer (day 3) were conducted. The good quality embryo was defined as 6–8 cells with less than 20% fragments. Ultrasound scans examination was performed at 7–8 weeks gestation to determine the clinical pregnancy outcome. The hormonal measurement results were analyzed using medical records of patients tested at day 2–3 of menstrual cycle.

### Genotyping

Genomic DNA sample was extracted from peripheral blood leukocytes by the method of phenol-chloroform extraction. Polymerase chain reaction (PCR) restriction fragment length polymorphism (RFLP) was used to determine the genotypes of *TP*53 codon 72 polymorphism and *HDM*2 SNP 309 polymorphism. All DNA samples were stored at -20°C before analysis. A random 10% of samples were repeated to verify the genotyping results. All the procedures were performed in the Laboratory of Molecular Genetics of Aging & Tumor, Faculty of Medicine, Kunming University of Science and Technology. The genotypes were assigned using all of the data from the study.

For the *TP*53 codon 72 polymorphism, the forward primer sequence 5’-AGC AGA GAC CTG TGG GAA GCG A and reverse primer 5’-CAG GGC AAC TGA CCG TGC AAG T were used to generate a 473bp fragment. The general PCR conditions were: initial denaturation at 94°C for 5 minutes, 35 cycles of denaturation at 94°C for 30 seconds, annealing at 60°C for 30 seconds, polymerization at 72°C for 30 seconds, and the final extension at 72°C for 5 minutes. The products were digested by the restriction enzyme BstUI (New England Biolabs, Beverly, MA, USA) at 60°C for 20 hours. The fragment with G allele was cut into 309bp and 164 bp, but the fragment with C allele was undigested (473bp). The digested solution was loaded into 2% agarose gel containing ethidium bromide for electrophoresis.

For the *HDM*2 SNP 309, The forward primer sequence 5’-CGC GGG AGT TCA GGG TAA AG and reverse primer 5’- AGC TGG AGA CAA GTC AGG ACT TAA C were used to amplify a 236bp fragment. The amplified products were digested overnight by the restriction enzyme MspA1I (New England Biolabs, Beverly, MA, USA). The fragment with GG allele was cut into 186bp and 50 bp, but the fragment with TT allele was undigested (236bp). The digested solution was loaded into 3% agarose gel containing ethidium bromide for electrophoresis to identify genotype.

### Statistical analysis

Data analysis was conducted using SPSS 15.0 software, The Chi-square test or Fisher's exact test was used to calculate the Hardy-Weinberg equilibrium (HWE) in healthy controls to exclude the possible bias during the selection of controls, as well as to compare the genotype and allele frequencies between the case and control groups. We used the Student’s t-test and Chi-square test to analyze the demographic and clinical characteristics of the case and control samples. All the values were presented as means. The adjusted odds ratios (ORs) and 95% confidence intervals (CIs) were also used by a multiple logistic regression. In the two-tailed test, a probability level of <0.05 was defined as a statistically significant result. For multiple comparisons among the combination of two genes’s polymorphisms, totally 9 subgroups were formed, p<0.0056(0.05/9) was defined as a statistically significant result based on the principle of Bonferroni correction. To avoid the assumptions of genetic models, dominant, recessive and allele models for *TP*53 codon 72 polymorphism were also evaluated. And the Kruskal-Wallis nonparametric one-way analysis of variance (ANOVA) test was conducted to determine differences between groups for quantitative variables.

## Results

A total of 1450 cases and 250 controls were included in the study and all genotypes of participants were detected successful. The mean ages of cases and controls were 31.82±4.40 and 30.24±5.61 at recruitment respectively. Case and control groups are all meet the Hardy Weinberg Equilibrium (HWE) considering the distribution of *TP*53 codon 72 and *HDM*2 SNP309 polymorphic genotypes (p = 0.29, p = 0.16). The IVF outcome of different genotypes was adjusted by age and embryo number of ET.

### *TP53* codon 72 polymorphism and *HDM*2 SNP 309 polymorphism with risk of infertility

The frequencies of *TP53* codon 72 polymorphism and *HDM*2 SNP309 polymorphism were presented in Tables 1 and 2. The results showed that no significant difference was found in different genetic models between infertility and control groups in view of *TP*53 codon 72 polymorphism and *HDM*2 SNP309 polymorphism.

**Table 1:** Association of *TP53* codon72 polymorphism and female infertility.

	Cases(n = 1450)		Controls(n = 250)		OR(95%CI)	*p*^*a*^
	n	%	n	%		
Codominant						
G/G	362	24.97	65	26.00	1	
C/G	747	51.52	133	53.20	0.99(0.73–1.39)	0.96
C/C	341	23.52	52	20.80	1.18(0.77–1.65)	0.42
Recessive						
G/G +C/G	1109	76.48	198	79.20	1	
C/C	341	23.52	52	20.80	1.18(0.75–1.53)	0.35
Dominant						
G/G	362	24.97	65	26.00	1	
C/C+C/G	1088	75.03	185	74.00	1.06(0.78–1.55)	0.73
Allele						
G	1471	50.72	263	52.60	1	
C	1429	49.28	237	47.40	1.08(0.79–1.31)	0.44

*p*^*a*^: adjusted p value for age

**Table 2:** Association of *HDM*2 SNP309 polymorphism and female infertility.

*HDM*2	Cases(n = 1450)		Controls(n = 250)		OR(95%CI)	*p*^*a*^
	n	%	n	%		
Codominant						
G/G	355	24.48	65	26.00	1	
T/G	778	53.66	139	55.60	0.98(0.74–1.41)	0.62
T/T	317	21.86	46	18.40	0.79(0.54–1.60)	0.37
Dominant						
G/G+T/G	1133	78.14	204	81.60	1	
T/T	317	21.86	46	18.40	1.23 (0.68–1.55)	0.46
Recessive						
G/G	355	24.48	65	26.00	1	
T/T+T/G	1095	75.52	185	74.00	1.08(0.82–1.52)	0.55
Allele						
G	1489	51.34	269	53.80	1	
T	1411	48.66	231	46.20	1.06 (0.81–1.34)	0.39

*p*^*a*^: adjusted p value for age

### *TP*53 codon 72 polymorphism and *HDM*2 SNP 309 polymorphism with IVF outcome

A total of 1449 women undergoing their first IVF treatment cycles were enrolled in the analysis. Of the 1449 patients, 569 were positive and 880 were negative clinical pregnancy outcome. We analyzed the demographic characteristics and clinical profiles related to pregnancy such as patient mean age, body mass index, duration of infertility, basal FSH levels, basal LH levels, thickness of endometrium, number of oocytes retrieved, number of fertilized oocytes, number of cleavage embryos, number of transferred embryos and number of good quality embryos among different genotypes of *TP*53 codon 72 polymorphism and *HDM*2 SNP309 polymorphism (shown in Tables 3 and 4), and no significant difference was found.

**Table 3:** Basal information of patient with different *TP53* codon72 SNP.

	C/C	C/G	G/G	p
Age(years)	31.67±4.17	31.94±4.41	31.70±4.60	0.55
Body Mass Index	21.69±3.34	21.90±4.87	22.18±5.99	0.42
Duration of infertility(years)	4.78±3.43	4.97±3.41	5.04±3.52	0.57
FSH(IU/L)	6.02±3.58	6.30±3.30	6.33±3.53	0.77
Thickness of endometrium (day of hCG)	9.05±4.63	8.95±5.13	8.78±4.83	0.32
LH(IU/L)	5.77±3.10	7.21±3.33	6.09±3.86	0.60
Number of oocytes retrieved	8.87±6.46	8.90±6.49	9.04±6.54	0.93
Number of fertilized oocytes	6.18±4.21	6.26±4.45	6.14±4.28	0.91
Number of cleavage embryos	6.03±4.15	6.15±4.39	6.03±4.24	0.86
Number of transferred embryos	2.12±0.54	2.16±0.55	2.13±0.56	0.57
Number of good quality embryos	3.37±2.85	3.44±2.99	3.17±2.93	0.38

**Table 4:** Basic information of female patients with different *HDM*2 SNP309.

	T/T	T/G	G/G	p
Age(years)	32.08±4.66	32.12±4.72	32.50±4.65	0.33
Body Mass Index	22.20±4.89	22.20±5.49	21.78±4.26	0.36
Duration of infertility(years)	5.00±3.28	4.86±3.48	5.20±3.75	0.27
FSH(IU/L)	6.17±4.60	6.15±6.94	6.30±5.39	0.91
Thickness of endometrium (day of hCG)	6.07±6.65	5.93±7.49	7.67±4.23	0.41
LH(IU/L)	8.99±5.08	8.62±5.03	8.90±5.04	0.43
Number of oocytes retrieved	8.56±6.24	9.17±6.59	8.45±6.08	0.11
Number of fertilized oocytes	5.88±4.06	6.39±4.42	5.90±4.19	0.07
Number of cleavage embryos	2.16±0.59	2.17±0.55	2.15±0.58	0.84
Number of transferred embryos	3.15±2.75	3.35±2.87	3.41±3.15	0.43

The distributions of *TP*53 codon 72 polymorphism between negative and positive groups were shown in Table 5. The C allele showed a higher frequency in positive clinical pregnancy group than the negative group (52.10% vs. 47.40%, p = 0.01). A strong significant association was found between C allele and IVF outcome (OR = 0.83, 95%CI:0.71–0.96, *p* = 0.01) with adjusted p value by age and embryo’s number of ET for their vital impact roles in embryo implantation, which suggested that C allele decreased the risk of pregnancy failure after IVF.

**Table 5:** Frequencies of female *TP53* codon72 polymorphism among women with different IVF outcome.

Codominant	IVF outcome				OR(95%CI)	*p*a
	negative		positive			
	n	%	n	%		
G/G	234	26.59	128	22.50	1	
C/G	458	52.05	289	50.79	0.86(0.67–1.10)	0.52
C/C	188	21.36	152	26.71	0.65(0.49–0.87)	0.02
Recessive						
G/G+C/G	692	78.64	417	73.29	1	
C/C	188	21.36	152	26.71	0.72(0.57–0.91)	0.02
Dominant						
G/G	234	26.59	128	22.50	1	
C/C+C/G	646	73.41	441	77.50	0.79(0.63–0.99)	0.17
Allele						
G	926	52.61	545	47.89	1	
C	834	47.39	593	52.11	0.83(0.71–0.96)	0.01

*p*^*a*^: adjusted p value for age and embryo number of ET

No significant difference was found among both groups for *HDM*2 SNP309 polymorphic genotypes (shown in Table 6). It means that *HDM*2 SNP309 polymorphism is not associated with pregnancy outcome of IVF.

**Table 6:** Association of female *HDM*2 SNP309 and IVF outcome.

Codominant	IVF outcome				OR(95%CI)	*p*^a^
	negative		positive			
	n	%	n	%		
G/G	223	0.2534	131	0.2302	1	
T/G	467	0.5307	311	0.5466	0.88(0.64–1.20)	0.29
T/T	190	0.2159	127	0.2232	0.99(0.76–1.30)	0.76
Recessive						
G/G+T/G	690	0.7841	442	0.7768	1	
T/T	190	0.2159	127	0.2232	0.80(0.58–1.33)	0.53
Dominant						
T/T+T/G	657	0.7466	438	0.7698	1	
G/G	223	0.2534	131	0.2302	0.88(0.69–1.13)	0.26
Allele						
G	914	0.5193	573	0.5035	1	
T	846	0.4807	565	0.4965	0.93(0.82–1.14)	0.3

*p*^*a*^: adjusted p value for age and embryo number of ET

### The combination of *TP53* codon 72 polymorphism and *HDM*2 SNP 309 polymorphism with IVF outcome

The effect of combination between *TP53* codon 72 polymorphism and *HDM*2 SNP 309 polymorphism on IVF outcome are listed in Table 7. We carried out a Bonferroni correction for multiple statistical tests, p<0.0056 was defined as a statistically significant difference. Of 9 combined forms, no significant differences were found in clinical pregnancy rate among different combination forms compared to all the other remained forms.

**Table 7:** Conjoint analysis between pregnancy outcome and female polymorphism of *TP53* codon72 and *HDM*2 SNP309.

*TP*53codon72SNP	*HDM*2 SNP309	numbers	Pregnancy rate	OR(95%CI)	Mean ages	p^a^
C/C	T/T	75	44.00%	0.84(0.48–1.47)	31.92±3.87	0.57
C/C	T/G	175	44.57%	0.94(0.65–1.35)	31.70±4.41	0.82
C/C	G/G	90	45.56%	0.65(0.38–1.12)	31.42±3.94	0.16
C/G	T/T	155	40.00%	0.96(0.65–1.39)	31.50±4.42	0.93
C/G	T/G	414	38.89%	1.03(0.80–1.34)	32.01±4.42	0.87
C/G	G/G	178	37.08%	1.12(0.79–1.59)	32.17±4.37	0.58
G/G	T/T	86	37.21%	1.07(0.65–1.783)	31.58±4.78	0.91
G/G	T/G	190	37.89%	1.39(0.96–2.02)	31.16±4.23	0.21
G/G	G/G	86	27.91%	2.32(1.49–3.62)	33.02±4.97	0.04
Total		1449	39.27%		31.82±4.40	

*p*^*a*^: adjusted p value for age and embryo number of ET and p<0.0056(0.05/9) was defined as a statistically significant difference

## Discussion

The study explored polymorphisms of *TP*53 codon 72 and *HDM*2 SNP309 to obtain new insight about their association with human infertility and IVF outcome. Significant differences were revealed between allelic frequencies of *TP*53 codon 72 polymorphism and IVF outcome in women undergoing their first IVF cycles in the study. The distribution frequency of *TP*53 codon 72 C allele was higher in positive pregnancy patients after their first IVF cycle than negative pregnancy patients. The statistically significant differences were not found between infertility patients and controls for the distributions of two vital functional polymorphisms in apoptosis pathway genes Similarly, no significant difference was found between positive and negative pregnancy women undergoing IVF treatment for *HDM*2 SNP309 polymorphism and the combination with *TP*53 codon 72 polymorphism.

The result of our study was inconsistent with previous reports. It was demonstrated that C allele of *TP*53 codon 72 and G allele of *HDM*2 SNP309 polymorphism were enriched in IVF patients and C allele served as a risk factor of embryo implantation [14]. No significant difference was found in clinical pregnancy rate of IVF cycles with *TP*53 codon 72 polymorphism reported in another study by Patounakis et al [13]. Moreover, Lledo et al. revealed that *TP*53 gene codon 72 polymorphism in RIF and RPL patients is more prevalent than fertile controls; patients carrying a C homozygote genotype on *TP*53 codon 72 will have less chance to achieve an ongoing pregnancy after IVF[21]. These disagreements may result from the different selection of patients. In order to determine whether two functional SNPs of *TP* 53 and *HDM*2 have an influence on human female fertility and pregnancy rate after IVF and whether the SNPs is more prevalent in infertile population, our study selected the general infertility population in IVF center and fertile women as control. Therefore we did not assess other possible relationship between the polymorphisms and infertility factors. The study of Kang et al. just enrolled the unexplained infertility IVF patients may limit the association of the special population with *TP*53 codon 72 and *HDM*2 SNP309 polymorphisms; moreover, the reduction of pregnancy rate after IVF for C allele was confined to this unexplained infertility group[14]. This may be hard to elucidate the real effect of *TP*53 and *HDM*2 important polymorphisms on human reproduction. Another possible explanation about different results is the genetic heterogeneity. It was published that different distributions for the polymorphisms of *TP*53 codon 72 and *HDM*2 SNP309 in different regional or ethnic populations [22, 23]. The functional polymorphisms of *TP*53 and *HDM*2 may derive from selective pressure from different environmental exposures [24]. Moreover, *TP*53 functional polymorphism or other polymorphism might play a subtle role in human fecundity, which can be reflected only in larger sample size studies.

But Patounakis et al. reported that no enrichment of C allele of TP53 codon 72 was found in IVF patients with agreement with our results [15]. Patounakis et al. selected all patients without exclusion criteria, for male infertility, most female partners are fertile, which may become the confounding factor to the result. With respect to *TP*53 codon 72 polymorphism, most of studies about human reproduction focused on RIF and/or RPL women to draw some conflict conclusions[11, 12, 15, 21, 25]. Excepting for the selection of studied subjects, ethnic and regional discrepancies may contribute to these inconsistent results.

Successful pregnancy mainly depends on some intricate events which remain ambiguity such as appropriate proliferation, invasion into endometium and angiogenesis. Even though, some signals regulating embryo development and uterine receptivity are generated necessarily to support these processes. *TP*53 gene affects these events and *TP*53 codon 72 polymorphism may alter these processes resulting in implantation failure or very early pregnancy loss [11]. It is well known that C72 has been shown to induce apoptosis lower than G72 and C72 is associated with enhanced proliferation and implantation potential. These functions of C72 perhaps were performed during the early stage of pregnancy or around the time of implantation[12], which may explain our results that C allele raises the pregnancy rate of IVF. In addition, G allele has been found to induce apoptosis more than C72 [10], it may initiate the apoptosis procedure around the peri-implantation period responding to some exogenous environmental stresses for IVF treatment partly involves in several anthropogenic programs such as controlled ovary hyperstimulation, oocyte aspiration, in vitro fertilization-embryo culture and embryo transfer et al. Thus, G allele might decrease the IVF outcome to some extent. That can be accounted for the gene-environment interaction resulting in different susceptibility to different environmental factor even with the same polymorphic genotype.

*TP*53 regulates maternal reproduction at implantation stage through controlling its target gene leukemia inhibitory factor (LIF), LIF is a crucial cellular factor during blastocyst implantation. *TP*53 regulates a transiently increased expression of LIF in uterus which is accordant to the onset of implantation [8]. In view of LIF and embryo implantation, the result of our study disagrees with that mechanism. A plausible explanation is that *TP*53 performs its apoptosis role responding to some cell exposures surrounding early stage of implantation before the recognition of conception in spite of occurrence of implantation. Human reproduction was regulated by many genes and gene network and affected by different influence factors, how these genes interact with these factors to participate in and complete the procedure remains unknown. Many further studies need to be done to illustrate the procedure.

In summary, the results supported the association *TP*53 codon 72 polymorphism with IVF outcome. In other words, which means the carriers of C allele should have higher chance to be pregnant than the others. The limitation of the study was the lack of mechanic explanation, the subgroup analysis based on the infertility causes, lack the information of the subsequent IVF outcome for negative patients after their first ET cycles, lack the embryo related genotypes analysis and fewer SNPs of genes analyzed in *TP*53 pathway. Other mutations of *TP*53 pathway need to be searched, as it has been shown to be essential in apoptosis and angiogenesis during the normal pregnancy development. Detailed further studies with a larger sample size need to be performed to confirm our results. Meantime, it is also important that related mechanical studies in vivo should be carried out to testify the biological processes and associations.

## Supporting Information

S1 File This is the STROBE Checklist. (DOC)
