# Supplementary material for: Influence of TP53 Codon 72 Polymorphism Alone or in Combination with HDM2 SNP309 on Human Infertility and IVF Outcome
Source: PLoS One. 2016 Nov 29;11(11):e0167147. doi: 10.1371/journal.pone.0167147 (PMC5127557; doi:10.1371/journal.pone.0167147)
Supplement: S1 File — (DOC) [file pone.0167147.s001.doc]

STROBE Statement—checklist of items that should be included in reports of observational studies

|  | | | | Item No. | | Recommendation | | | Page  No. | | Relevant text from manuscript | | | |
| --- | --- | --- | --- | --- | --- | --- | --- | --- | --- | --- | --- | --- | --- | --- |
| **Title and abstract** | | | | 1 | | (*a*) Indicate the study’s design with a commonly used term in the title or the abstract | | | 2 | | Case-control study | | | |
|  |  |  |  |  |  | (*b*) Provide in the abstract an informative and balanced summary of what was done and what was found | | | 2 | | To evaluate the association of the TP53 codonalone or in combination with HDM2 SNP309 polymorphisms with human infertility and IVF outcome. C allele is a protective factor and combination of G/G (TP53) and G/G (HDM2) genotype is a risk factor for IVF pregnancy outcome. | | | |
| Introduction | | | | | | | | | | |  | | | |
| Background/rationale | | | | 2 | | Explain the scientific background and rationale for the investigation being reported | | | 3-4 | | TP53 participates in human reproduction. TP53 and HDM2 form an autoregulatory negative feedback loop, TP53 codon 72 polymorphism is associated with human fertility. | | | |
| Objectives | | | | 3 | | State specific objectives, including any prespecified hypotheses | | | 3-4 | | The aim of the presented study was to define the possible impact of TP53 codon 72 polymorphism (rs1042522) and/or HDM2 SNP 309(rs2279744) on human fertility and the first IVF pregnancy outcome. | | | |
| Methods | | | | | | | | | | |  | | | |
| Study design | | | | 4 | | Present key elements of study design early in the paper | | | 5-7 | | The case-control study, Subjects, Genotyping, Statistical analysis | | | |
| Setting | | | | 5 | | Describe the setting, locations, and relevant dates, including periods of recruitment, exposure, follow-up, and data collection | | | 5 | | All participants were recruited from The First People’s Hospital of Yunnan Province. Blood samples were collected from July 2010 to December 2012. | | | |
| Participants | | | | 6 | | (*a*) *Cohort study*—Give the eligibility criteria, and the sources and methods of selection of participants. Describe methods of follow-up  *Case-control study*—Give the eligibility criteria, and the sources and methods of case ascertainment and control selection. Give the rationale for the choice of cases and controls  *Cross-sectional study*—Give the eligibility criteria, and the sources and methods of selection of participants | | | 5 | | The infertility group consists of 1450 patients undergoing their first IVF cycle, the exclusion criteria are as follows. The control group consists of 250 women without infertility history. | | | |
|  |  |  |  |  |  | (*b*)*Cohort study*—For matched studies, give matching criteria and number of exposed and unexposed  *Case-control study*—For matched studies, give matching criteria and the number of controls per case | | |  | | N/A | | | |
| Variables | | | | 7 | | Clearly define all outcomes, exposures, predictors, potential confounders, and effect modifiers. Give diagnostic criteria, if applicable | | | 7 | | to compare the genotype and allele frequencies between the case and control groups, to analyze the demographic and clinical characteristics of the case and control samples. | | | |
| Data sources/measurement | | | | 8* | | For each variable of interest, give sources of data and details of methods of assessment (measurement). Describe comparability of assessment methods if there is more than one group | | | 7 | | PCR-RFLP was used to determine the genotypes of TP53 codon 72 polymorphism (rs 1042522) and HDM2 SNP 309 (rs2279744) polymorphism. clinical pregnancy outcome | | | |
| Bias | | | | 9 | | Describe any efforts to address potential sources of bias | | | 8 | | Data analysis was conducted using SPSS 15.0 software, The Chi-square test or Fisher's exact test was used to calculate the Hardy-Weinberg equilib­rium (HWE) in healthy controls to exclude the possible bias during the selection of controls. | | | |
| Study size | | | | 10 | | Explain how the study size was arrived at | | | 5 | | The sample size was evaluated for the genotyped SNPs with the use of a relevant genotype frequency, The research was ceased once it reached the statistical significance. | | | |
| Quantitative variables | | 11 | | | Explain how quantitative variables were handled in the analyses. If applicable, describe which groupings were chosen and why | | | 8 | | | | And the Kruskal-Wallis nonparametric one-way analysis of variance (ANOVA) test was conducted to determine differences between groups for quantitative variables. | |  |
| Statistical methods | | 12 | | | (*a*) Describe all statistical methods, including those used to control for confounding | | | 7-8 | | | | The adjusted odds ratios (ORs) and 95% confidence intervals (CIs) were also used by a multiple logistic regression | |  |
|  |  |  |  |  | (*b*) Describe any methods used to examine subgroups and interactions | | | 7-8 | | | | We used the Student’s t-test and Chi-square test to analyze the demographic and clinical characteristics of the case and control samples. One-way analysis of variance (ANOVA) test was conducted to determine differences between groups for quantitative variables. | |  |
|  |  |  |  |  | (*c*) Explain how missing data were addressed | | |  | | | | N/A | |  |
|  |  |  |  |  | (*d*) *Cohort study*—If applicable, explain how loss to follow-up was addressed  *Case-control study*—If applicable, explain how matching of cases and controls was addressed  *Cross-sectional study*—If applicable, describe analytical methods taking account of sampling strategy | | |  | | | | N/A | |  |
|  |  |  |  |  | (*e*) Describe any sensitivity analyses | | |  | | | | N/A | |  |
| Results | | | | | | | | | | | | | |  |
| Participants | | 13* | | | (a) Report numbers of individuals at each stage of study—eg numbers potentially eligible, examined for eligibility, confirmed eligible, included in the study, completing follow-up, and analysed | | | 8- | | | | A total of 1450 cases and 250 controls were included in the study and all genotypes of participants were detected successful. Case and control groups are all meet the Hardy Weinberg Equilibrium (HWE). | |  |
|  |  |  |  |  | (b) Give reasons for non-participation at each stage | | |  | | | | N/A | |  |
|  |  |  |  |  | (c) Consider use of a flow diagram | | |  | | | | N/A | |  |
| Descriptive data | | 14* | | | (a) Give characteristics of study participants (eg demographic, clinical, social) and information on exposures and potential confounders | | | 8，10 | | | | The mean ages of cases and controls were 31.82±4.40 and 30.24±5.61 at recruitment respectively. We analyzed the demographic characteristics and clinical profiles related to pregnancy such as patient mean age, body mass index, duration of infertility, basal FSH levels, basal LH levels, thickness of endometrium, number of oocytes retrieved, number of fertilized oocytes, number of cleavage embryos, number of transferred embryos and number of good quality embryos | |  |
|  |  |  |  |  | (b) Indicate number of participants with missing data for each variable of interest | | |  | | | | N/A | |  |
|  |  |  |  |  | (c) *Cohort study*—Summarise follow-up time (eg, average and total amount) | | |  | | | | N/A | |  |
| Outcome data | | 15* | | | *Cohort study*—Report numbers of outcome events or summary measures over time | | |  | | | | N/A | |  |
|  |  |  |  |  | *Case-control study—*Report numbers in each exposure category, or summary measures of exposure | | | 8-14 | | | | TP53 codon 72 polymorphism and HDM2 SNP 309 polymorphism with risk of infertility. TP53 codon 72 polymorphism (rs1042522) and HDM2 SNP 309 (rs 2279744) polymorphism with IVF outcome. The combination of TP53 codon 72 polymorphism (rs1042522) and HDM2 SNP 309 (rs 2279744) polymorphism with IVF outcome. | |  |
|  |  |  |  |  | *Cross-sectional study—*Report numbers of outcome events or summary measures | | |  | | | | N/A | |  |
| Main results | | 16 | | | (*a*) Give unadjusted estimates and, if applicable, confounder-adjusted estimates and their precision (eg, 95% confidence interval). Make clear which confounders were adjusted for and why they were included. | | | 8-14 | | | | We adjusted the P value directly for the absolute role during the period of embryo implantation of maternal age and embryo number of ET. | |  |
|  |  |  |  |  | (*b*) Report category boundaries when continuous variables were categorized | | |  | | | | N/A | |  |
|  |  |  |  |  | (*c*) If relevant, consider translating estimates of relative risk into absolute risk for a meaningful time period. | | |  | | | | N/A | |  |
| Other analyses | 17 | | Report other analyses done—eg analyses of subgroups and interactions, and sensitivity analyses | | | | 13-14 | | | The effect of combination between TP53 codon 72 (rs1042522) polymorphism and HDM2 SNP 309 (rs 2279744) polymorphism on IVF outcome are listed in Table 7. | | |  | |
| Discussion | | | | | | | | | | | | |  | |
| Key results | 18 | | Summarise key results with reference to study objectives | | | | 14 | | | The study explored polymorphisms of TP53 codon 72 (rs1042522) and HDM2 SNP309 (rs 2279744) to obtain new insight about their association with human infertility and IVF outcome. Significant differences were revealed between allelic frequencies of TP53 codon 72 (rs1042522) polymorphism alone or in combination with HDM2 SNP309 (rs 2279744) polymorphism and IVF outcome in women undergoing their first IVF cycles in the study. | | |  | |
| Limitations | 19 | | Discuss limitations of the study, taking into account sources of potential bias or imprecision. Discuss both direction and magnitude of any potential bias | | | | 18 | | | The study explored polymorphisms of TP53 codon 72 (rs1042522) and HDM2 SNP309 (rs 2279744) to obtain new insight about their association with human infertility and IVF outcome. Significant differences were revealed between allelic frequencies of TP53 codon 72 (rs1042522) polymorphism alone or in combination with HDM2 SNP309 (rs 2279744) polymorphism and IVF outcome in women undergoing their first IVF cycles in the study. | | |  | |
| Interpretation | 20 | | Give a cautious overall interpretation of results considering objectives, limitations, multiplicity of analyses, results from similar studies, and other relevant evidence | | | | 15-17 | | | Pregnancy is a complicated issue, the diversified interactions between TP53 and HDM2 polymorphism genotypes possibly were associated with IVF outcome. However, the precise mechanism was known little until now. | | |  | |
| Generalisability | 21 | | Discuss the generalisability (external validity) of the study results | | | | 18 | | | C allele and combination of G/G (*TP*53) and G/G (*HDM*2) should have higher chance to be pregnant than the others. | | |  | |
| Other information | | |  | | | | | | | | | |  | |
| Funding | 22 | | Give the source of funding and the role of the funders for the present study and, if applicable, for the original study on which the present article is based | | | | 19 | | | The study was supported by grants GREKF10-07 from the Fund of State Key Laboratory of Genetics Resources and Evolution. | | |  | |

*Give information separately for cases and controls in case-control studies and, if applicable, for exposed and unexposed groups in cohort and cross-sectional studies.

**Note:** An Explanation and Elaboration article discusses each checklist item and gives methodological background and published examples of transparent reporting. The STROBE checklist is best used in conjunction with this article (freely available on the Web sites of PLoS Medicine at http://www.plosmedicine.org/, Annals of Internal Medicine at http://www.annals.org/, and Epidemiology at http://www.epidem.com/). Information on the STROBE Initiative is available at www.strobe-statement.org.
